# Transferrin receptor-targeting property of pabinafusp alfa facilitates its uptake by various types of human brain-derived cells *in vitro*

**DOI:** 10.3389/fddev.2023.1082672

**Published:** 2023-07-03

**Authors:** Tomoki Fukatsu, Hanae Morio, Tomomi Furihata, Hiroyuki Sonoda

**Affiliations:** ^1^ JCR Pharmaceuticals, Research Division, Kobe, Japan; ^2^ Laboratory of Clinical Pharmacy and Experimental Therapeutics, School of Pharmacy, Tokyo University of Pharmacy and Life Sciences, Tokyo, Japan

**Keywords:** mucopolysaccharidosis II, pabinafusp alfa, iduronate-2-sulfatase, blood-brain barrier, transferrin receptor, cellular uptake, transcytosis

## Abstract

Pabinafusp alfa, which is an anti-mucopolysaccharidosis II drug, consists of iduronate-2-sulfatase (IDS) genetically fused with an anti-transferrin receptor (TfR) antibody. While IDS is known to enter cells via mannose-6-phosphate receptor (M6PR)-mediated endocytosis, the anti-TfR antibody moiety of pabinafusp alfa is supposed to trigger the TfR-mediated transcytosis involved in its blood-brain barrier (BBB) penetration to deliver IDS into the brain, which thus makes it effective for treatment of brain symptoms of the disease. However, since these uptake processes remain unexamined *in vitro*, this study aims at elucidating how human brain cells manipulate these receptors to facilitate pabinafusp alfa uptake. The results of pabinafusp alfa uptake assays showed that the TfR played an primary role in its uptake by brain microvascular endothelial cells. The TfR contribution was also found in neuronal cells at levels comparable to M6PR. Interestingly, the predominant roles of TfR over M6PR in pabinafusp alfa uptake were also observed in astrocytes and pericytes. To summarize, our results support the TfR-targeting strategy of pabinafusp alfa for facilitating its BBB penetration while simultaneously identifying previously unnoticed TfR roles in its uptake into human neuronal and non-neuronal brain cells. These findings are certain to provide important insights into the mechanisms behind clinical actions of pabinafusp alfa.

## Introduction

Mucopolysaccharidosis II (MPS II) is a congenital dysmetabolic syndrome caused by the deficiency or decreased activity of iduronate-2-sulfatase (IDS), which is an enzyme responsible for the catabolism of the glycosaminoglycans (GAGs). While all MPS II patients experience progressive lysosomal GAG accumulation throughout their bodies, approximately 70% of those patients suffer from severe forms of the disease with remarkable central nervous system (CNS)-related manifestations, such as sleep apnea, seizures, and acute intellectual disability (Stapleton et al., 2017; Bigger et al., 2018; Zhou et al., 2020). Current MPS II treatment regimens include enzyme replacement therapy (ERT) and hematopoietic stem cell transplantation, both of which have been developed to replace recombinant IDS. However, one of the critical limitations of these therapies is that, while effective in ameliorating the peripheral symptoms, they do not treat CNS manifestations (Whiteman and Kimura, 2017; Concolino et al., 2018; Stapleton et al., 2019; Taylor et al., 2019). One of the primary causal factors behind this problem lies in the blood-brain barrier (BBB), which is formed by brain microvascular endothelial cells (BMECs) together with surrounding brain cells such as astrocytes and pericytes (Abbott et al., 2010; Abbott, 2013; Sharif et al., 2018). The BBB is a tight physical/biological obstacle that restricts the transportation of various substances into the brain. Therefore, to effectively treat CNS symptoms of MPS II, it is crucial to develop a specific delivery system via which therapeutic agents can effectively cross the BBB to reach the brain parenchyma.

**FIGURE 1:**
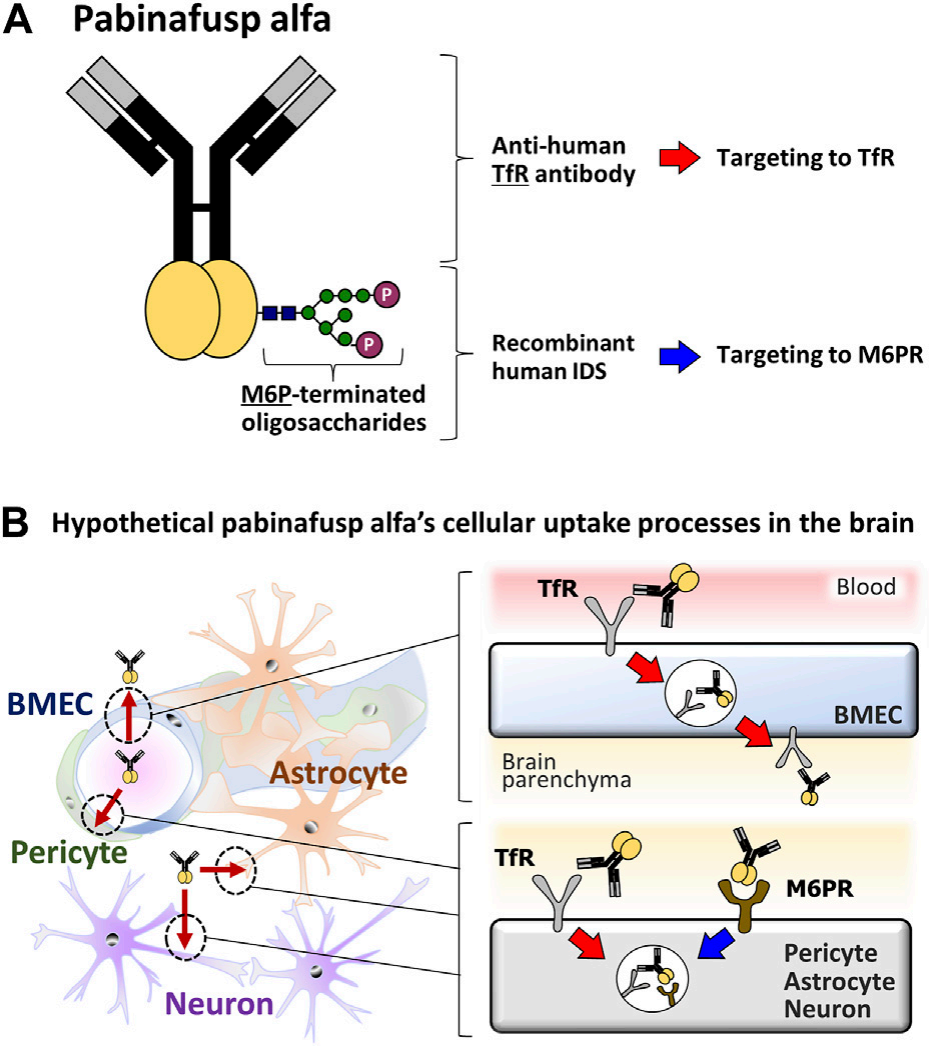
Diagrams of the pabinafusp alfa structure and its hypothetical cellular uptake routes. **(A)** The structure of pabinafusp alfa. Pabinafusp alfa consists of the anti-human TfR antibody moiety and the recombinant human IDS moiety (a main body exerting its therapeutic effects), which are genetically fused. The former part can bind to TfR, while the latter part, which carries a polysaccharide chain containing M6P, can recognize M6PR. **(B)** The hypothetical uptake routes of pabinafusp alfa in various types of the brain cells. Utilizing the anti-human TfR antibody moiety, pabinafusp alfa is supposed to pass through BMECs via TfR-mediated transcytosis. Then, pabinafusp alfa is assumed to be incorporated into various types of brain cells through M6PR-mediated endocytosis, as with the case of IDS. In these processes, its anti-human TfR antibody moiety may play additional roles by triggering TfR-mediated endocytosis.

Meanwhile, pabinafusp alfa, which is a recently approved drug consisting of IDS genetically fused with an anti-human transferrin receptor (TfR) antibody (Figure 1A), has proven to be a breakthrough advance in MPS II treatments, and several human clinical trials have shown its remarkable efficacy at improving CNS symptoms in patients (Giugliani et al., 2021; Okuyama et al., 2021). In line with these clinical effects in humans, preceding pre-clinical *in vivo* experiments have identified apparently widespread brain distribution of the drug in mice and monkeys, as well as the reduction of brain GAG levels in MPS II model mice (Sonoda et al., 2018; Morimoto et al., 2021).

The key feature behind the clinical success of pabinafusp alfa is its unique anti-TfR antibody moiety (Figure 1A), which distinguishes it as a first-in-class BBB penetrating technology for delivering IDS into the brain through TfR-driven receptor-mediated transcytosis (RMT) (Sato and Okuyama, 2020; Kaplon et al., 2022) that physiologically serves as a supply route of transferrin (Tf) into the brain. It has been considered likely that, upon binding to TfR on the luminal surface of BMECs, pabinafusp alfa undergoes endocytosis together with TfR before moving intracellularly to the opposite side, then eventually leaving the cell via exocytosis to the brain parenchyma (Friden et al., 1991; Tashima, 2020; Terstappen et al., 2021) (Figure 1B). After passing through the BBB, pabinafusp alfa is presumed to be incorporated into the brain cells via the mannose-6-phosphate receptor (M6PR) (Figure 1B), which recognizes M6P moieties appearing at the top of the IDS carbohydrate chains (Figure 1A).

However, the above-mentioned cellular uptake processes of pabinafusp alfa remain plausible hypotheses, and therefore they need to be thoroughly validated *in vitro*. In particular, it is intriguing to examine the potential roles of anti-TfR antibody moiety in its uptake into various types of brain cells since TfR can also be expressed therein. Therefore, with expectations of gaining further insights into the mode of action of pabinafusp alfa, this paper aims at proving the hypotheses by characterizing the roles played by the surface receptors in pabinafusp alfa uptake using different human brain cell types via comparisons with IDS.

## Materials and methods

### Cells and culture methods

Lund human mesencephalic (LUHMES) cell line (a dopaminergic neuronal cell line) was obtained from American Type Culture Collection (Manassas). The cells were routinely cultured in advanced Dulbecco’s Modified Eagle Medium (DMEM)/Nutrient Mixture F-12 (Thermo Fisher Scientific) supplemented with 1% (v/v) GlutaMAX (Thermo Fisher Scientific), 1% (v/v) N-2 supplement, recombinant basic fibroblast growth factor (40 ng/mL), and antibiotics. Culture dishes were coated with poly-L-ornithine (50 μg/mL) and fibronectin (1 μg/mL). For their differentiation, the cells were cultured in advanced DMEM/Nutrient Mixture F-12 supplemented with 1% (v/v) GlutaMAX, 1% (v/v) N-2 supplement, tetracycline (1 μg/mL), dibutyryl cyclic AMP (1 mM), and recombinant glial cell line-derived neurotrophic factor (2 ng/mL) (Zhang et al., 2014) for 2 days. Afterwards, the differentiated cells were seeded into 24-well plates with the same differentiation medium and cultured for another 4 days.

SH-SY5Y cell line was also obtained from American Type Culture Collection. The cells were routinely cultured in DMEM high glucose with 10% (v/v) fetal bovine serum and antibiotic, which was referred to as growth medium. For their differentiation, the two-step culture method was used (Forster et al., 2016). First, SH-SY5Y cells were cultured with the growth medium supplemented with retinoic acid (3 μg/mL) for 3 days, and in the second step, they were seeded into 24-well plates with Neurobasal-A medium minus phenol red (Thermo Fisher Scientific) containing glutamine (2 mM), 1% (v/v) N-2 supplement, and brain-derived neurotrophic factor (50 ng/mL) for another 4 days. Both neuronal cell lines were cultured at 37°C with 5% CO_2_/95% air.

Human BMEC/conditionally immortalized clone 18 (HBMEC/ci18), human astrocytes/conditionally immortalized clone 35 (HASTR/ci35) cells, and human brain pericytes/conditionally immortalized clone 37 (HBPC/ci37) cells, which have been developed in our previous studies (Furihata et al., 2016; Umehara et al., 2018; Ito et al., 2019), were cultured at 33°C as described therein except that the EGM-2 Bullet Kit (Lonza) was used for HBMEC/ci18 cell maintenance.

### Western blotting analyses

Cell lysates were prepared from the immortalized BBB cells and the differentiated neuronal cells by ultrasonication using RIPA buffer containing a protease inhibitor cocktail. Each lysate was mixed with 2×laemmli sample buffer, followed by incubation at 95°C for 3 min. The proteins (0.25 or 0.5 µg/lane) were separated by SDS-PAGE using a 5%–20% gradient gel and then transferred onto a nitrocellulose membrane. The membranes were blocked with 4% (w/v) Block ACE (KAC). The primary antibodies (Ab) used were: huTfR Ab (1 μg/mL) (Sonoda et al., 2018), rabbit anti-M6PR monoclonal Ab (50,000-fold dilution, ab124767, Abcam), rabbit anti-βIII tubulin polyclonal Ab (1,000-fold dilution, ab18207, Abcam), rabbit anti-microtubule-associated protein 2 (MAP2) polyclonal Ab (1,000-fold dilution, ab32454, Abcam), rabbit anti-tyrosine hydroxylase (TH) polyclonal Ab (1,000-fold dilution, AB152, Merck Millipore), and mouse anti-glyceraldehyde 3-phosphate dehydrogenase monoclonal IgG (1,000-fold dilution, FUJIFILM Wako Pure Chemical). The secondary antibodies used were anti-mouse IgG-horseradish peroxidase (HRP) conjugate (10,000-fold dilution, Promega) and anti-rabbit IgG-HRP conjugate (10,000-fold dilution, Promega). Immunocomplexes were detected using the Clarity Western ECL Substrate (BIORAD) and visualized by the Image Quant Imager 400 (GE Healthcare).

### Immunocytochemistry

Immunocytochemistry was used for confirmation of LUHMES and SH-SY5Y cell differentiation. After culturing with each differentiation protocol as described above, the cells were fixed with 4% paraformaldehyde/phosphate-buffered saline (PBS) (FUJIFILM Wako Pure Chemical) and permeabilized/blocked with 1% (w/v) bovine serum albumin/PBS-0.05% (w/v) Tween20 (hereafter referred as blocking buffer). The primary antibodies used were rabbit anti-βIII tubulin polyclonal Ab (1,000-fold dilution, ab18207, Abcam), rabbit anti-MAP2 polyclonal Ab (1,000-fold dilution, ab32454, Abcam), and rabbit anti-TH polyclonal Ab (250-fold dilution, AB152, Merck Millipore). The secondary antibody used was anti-rabbit IgG-Alexa Fluor 488 conjugate (2,000-fold dilution, #4412, Cell Signaling). All the antibodies were diluted to the indicated concentrations with the blocking buffer. For nuclear counter-staining, Hoechst33342 (Thermo Fisher Scientific) was used. The fluorescence was detected using the DMI 6000B (Leica).

#### Uptake assays

Pabinafusp alfa and IDS had been prepared in JCR Pharmaceuticals (Sonoda et al., 2018), and they were confirmed to carry carbohydrate chains. The ^125^I-labelling proteins were prepared with an iodogen method using iodogen tubes (Thermo Fisher Scientific) and used as substrates (1 μg/mL) in the uptake assays, which were performed in 24-well plates. huTfR Ab (10 μg/mL) and/or M6P (10 mM) was added to each substrate solution in the inhibition assays, in which human IgG isotype control (IgG, 10 μg/mL) (ab206198, Abcam) or glucose-6-phosphate (G6P, 10 mM) (G7879, Sigma Aldrich) was also used as a respective negative control. The substrate solution was added to the culture medium of the cells, followed by incubation for 1, 2 or 4 h. Afterwards, the cells were washed three-times with PBS(−) and then solubilized with 150 µL of the RIPA buffer. The aliquot of 10 µL of the cell lysates was applied to measurement of its radioactivity (cpm) using the Wallac Wizard1470 gamma counter (PerkinElmer). Protein quantification was conducted using a BCA Protein Assay Kit (Thermo Fisher Scientific) to normalize the radioactivity.

### *In vitro* BBB permeability assays

^125^I-labelled pabinafusp alfa and ^125^I-labelled IDS were prepared as described above and their permeability assays were performed using the HBMEC/ci18 monocultured BBB models. The models were prepared in a trans-well culture system according to the protocol shown in our previous report (Ito et al., 2019). Briefly, the membranes of cell culture inserts (translucent polyethylene terephthalate, 0.4 μm high-density pores, BD Falcon) were incubated with 100 μg/mL type-IV collagen (Nitta Gelatin)/100 μg/mL fibronectin (Sigma Aldrich) solution at 37°C for an hour. The inserts were dried by air and rinsed once with PBS(−), followed by seeding HBMEC/ci18 cells (1.3 × 10^5^ cells/cm^2^) onto the inner side of the coated insert membrane. In this seeding, the EGM-2 Bullet Kit (Lonza) without hVEGF, hEGF, and blastcidin S was used as the medium for both the inner and the outer sides of the insert.

One day after HBMEC/ci18 cell seeding, the medium was replaced with a new one and pre-incubated at 37°C for an hour. Either ^125^I-labelled pabinafusp alfa or ^125^I-labelled IDS (f.c. 1 μg/mL) was added to the apical side of the insert to start the assays, followed by incubation for 10, 20, 30, and 40 min (for pabinafusp alfa) or 5, 10, and 15 min (for IDS) at 37°C. The incubation time points of each drug were set to show the linear increase of their permeability levels. At each incubation time point, the medium was collected from the basolateral side of each insert. To determine the concentrations, the aliquot of 20 µL of the basolateral medium was applied to measurement of its radioactivity (cpm) using the Wallac Wizard1470 gamma counter (PerkinElmer). The permeability of each drug was evaluated by calculating the permeability-surface area product values (PS, µL/min) using a regression curve of the time-volume plot (the x- and *y*-axis, respectively).

### Statistical analyses

Statistical analyses were performed using data analysis tools of Microsoft Excel (Office 365) to determine whether the differences between the multiple values were significant. The values obtained from uptake assays were first analyzed by one-way ANOVA, followed by the Student’s t-test.

## Results

### A primary role of TfR in pabinafusp alfa uptake by human BMECs

The BBB-penetration ability of pabinafusp alfa, which is clear contrast to the BBB-impermeable property of IDS, serves a critical prerequisite role in its pharmacological actions in the brain. Therefore, we started this study by characterizing the pabinafusp alfa uptake pathways in HBMEC/ci18 cells that have shown a variety of BBB-specific characteristics (Ito et al., 2019; Kitamura et al., 2021).

**FIGURE 2:**
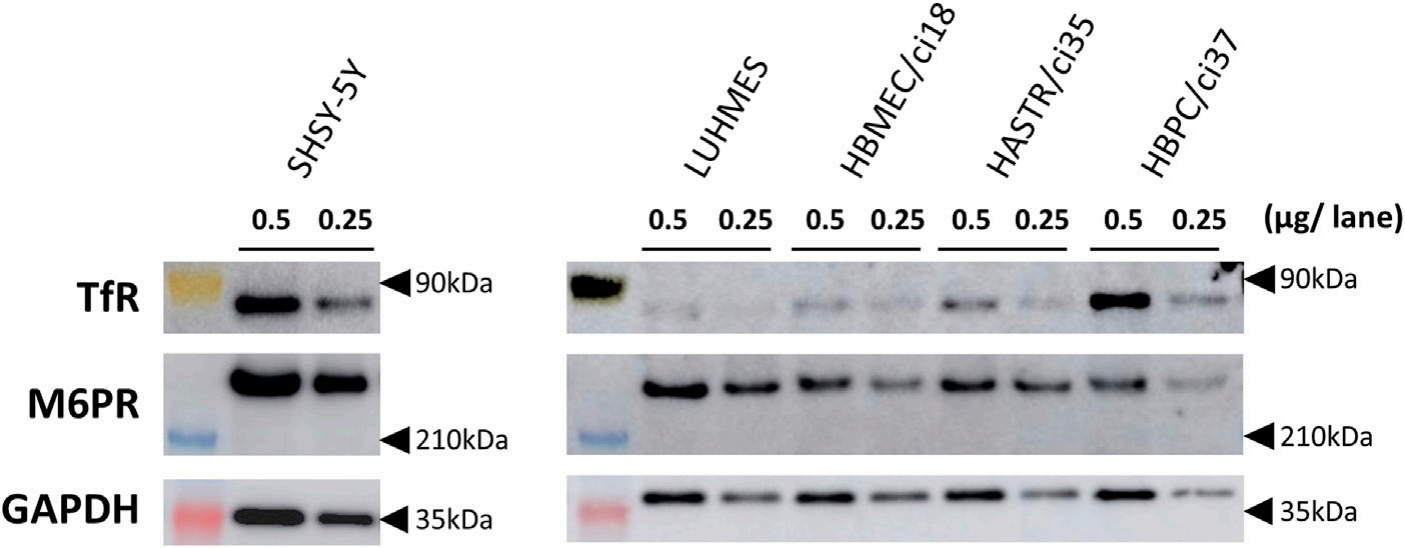
The protein expression of TfR and M6PR in different human brain-derived cell types. The protein expression of TfR and M6PR were examined by Western blotting analyses using whole cell lysates (0.25 or 0.5 μg/lane) prepared from differentiated SH-SY5Y, differentiated LUHMES, HBMEC/ci18, HASTR/ci35, and HBPC/ci37 cells. GAPDH was used as a loading control. The representative results obtained from two independent experiments (N = 2) are shown (uncropped images of Western blotting analyses are shown in Supplementary Figure S2).

**FIGURE 3:**
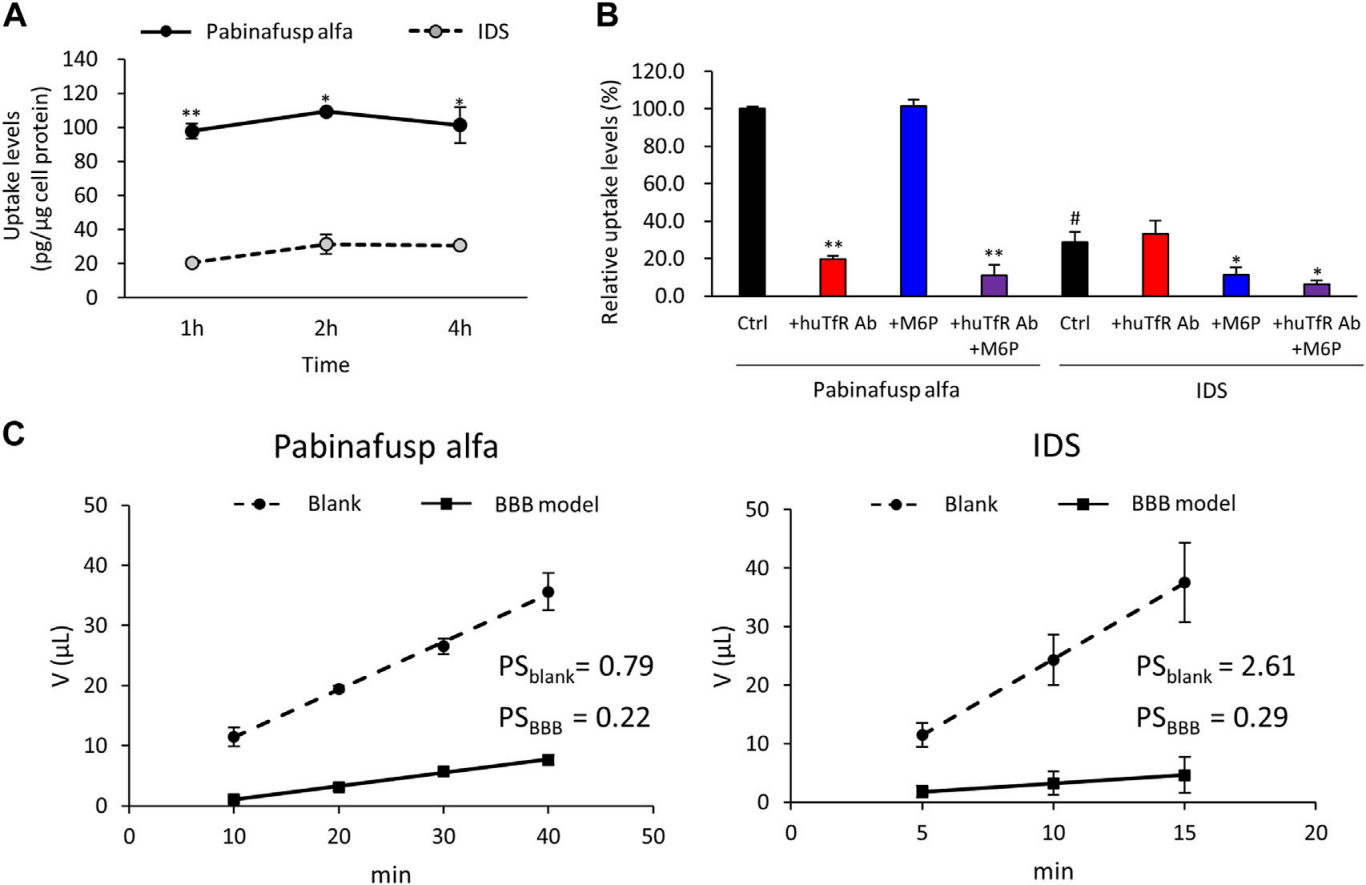
Characterization of pabinafusp alfa uptake profiles in BMECs. ^125^I-labeled pabinafusp alfa (1 μg/mL) or ^125^I-labeled IDS (1 μg/mL) uptake in HBMEC/ci18 and their *in vitro* BBB permeability were examined. **(A)** The incubation (uptake) periods were set at 1, 2, and 4 h, and the uptake levels (pg/μg cell protein) of pabinafusp alfa (solid line) and IDS (dotted line) are shown as the mean ± S.E. of values obtained from three separate experiments (N = 3), each performed in duplicate (*, *p* < 0.05; **, *p* < 0.01; Student’s t-test). The data of each experiment are shown in Supplementary Figure S3A. **(B)** The uptake levels were examined for 2 h in the presence or absence of huTfR Ab and/or M6P, which serve as competitive inhibitors for TfR and M6PR, respectively (huTfR Ab is the same anti-human TfR antibody as that used in pabinafusp alfa). Data shows the relative uptake level (%) when calculating the pabinafusp alfa uptake levels without inhibitors (Ctrl) as 100%. Data are shown as the mean ± S.E. of values obtained from three separate experiments (N = 3), each performed in duplicate (*, *p* < 0.05; **, *p* < 0.01 vs. each of the Ctrl; ^#^, *p* < 0.05 vs. pabinafusp alfa Ctrl; one-way ANOVA followed by Student’s t-test). The data of each experiment are shown in Supplementary Figure S3A. **(C)** The permeability experiments using HBMEC/ci18 mono-culture BBB models were conducted for calculating permeability-surface area product values (PS, µL/min) with the incubation periods set at 10, 20, 30, and 40 min for pabinafusp alfa and 5, 10, and 15 min for IDS. The volume (V) of compounds transmitted to the basolateral side (µL) in the BBB model (solid line) and the blank (dotted line) are shown as the mean ± S.E. of values obtained from three separate experiments (N = 3), each performed in duplicate. The PS values obtained from the BBB model and the blank (inserts without HBMEC/ci18 cells) are indicated as PS_BBB_ and PS_blank_, respectively. The data of each experiment are shown in Supplementary Figure S4.

Western blotting analyses were used to confirm the protein expression of the potential uptake receptors (*i.e.*, TfR and M6PR) in HBMEC/ci18 cells (Figure 2). The results pointed to the possible participation of M6PR in the pabinafusp alfa uptake in addition to TfR. Therefore, to see their relative contributions, we conducted uptake assays of pabinafusp alfa together with competitive inhibitors, which are M6P and a humanized anti-human TfR antibody (huTfR Ab, the same anti-human TfR antibody used in the pabinafusp alfa). The results showed that pabinafusp alfa was clearly taken up by HBMEC/ci18 cells (Figure 3A), which was significantly inhibited in the presence of huTfR Ab, but not by IgG (Supplementary Figure S3B) or M6P (Figure 3B). Hence, these results indicate practically no involvement of M6PR in pabinafusp alfa uptake into human BMECs, thereby highlighting the value of its TfR-targeting antibody moiety in facilitating BBB penetration.

We also performed IDS uptake experiments for a comparison purpose. As expected, the IDS uptake levels were considerably lower (Figure 3A), and they were sensitive to M6P, but not to huTfR Ab (Figure 3B).

Furthermore, we compared the BBB permeability levels between pabinafusp alfa and IDS using the HBMEC/ci18 monocultured BBB models. The models without HBMEC/ci18 cells were also prepared for a comparison (blank control). The results of the *in vitro* BBB permeability assays showed that the PS values (µL/min) of pabinafusp alfa were 0.22 ± 0.02 in the BBB models (PS_BBB_) and 0.79 ± 0.13 in the blank (PS_blank_), while those of IDS were 0.29 ± 0.05 (PS_BBB_), 2.61 ± 0.30 (PS_blank_), respectively (Figure 3C). Then we tentatively calculated the relative PS values by dividing the value obtained from the BBB models by those from the blank (PS_BBB_/PS_blank_), resulting in 0.28 and 0.11 for pabinafusp alfa and IDS, respectively. Therefore, the higher relative PS value of pabinafusp alfa indicates that it can be transferred to the brain side more effectively than IDS, which are consistent with the results shown in Figure 3A.

### Involvement of both TfR and M6PR in pabinafusp alfa uptake by human neuronal cells

After pabinafusp alfa passes through the BBB, the next step should be its incorporation into brain cells, especially neurons. While an M6PR-mediated uptake system is likely to be involved as expected from the literature (Gary-Bobo et al., 2007; Kanzaki et al., 2020), the TfR-targeting property of pabinafusp alfa may additionally contribute to the incorporation process. Therefore, we examined these two possibilities using two well-known human neuronal model cell lines (SH-SY5Y and LUHMES cells) (Scholz et al., 2011; Forster et al., 2016), in which both TfR and M6PR proteins were expressed as observed in Western blotting analyses (Figure 2). (Please note that, while they were used after differentiation throughout the study (as shown in Supplementary Figure S1), we simply refer to them as SH-SY5Y and LUHMES.)

**FIGURE 4:**
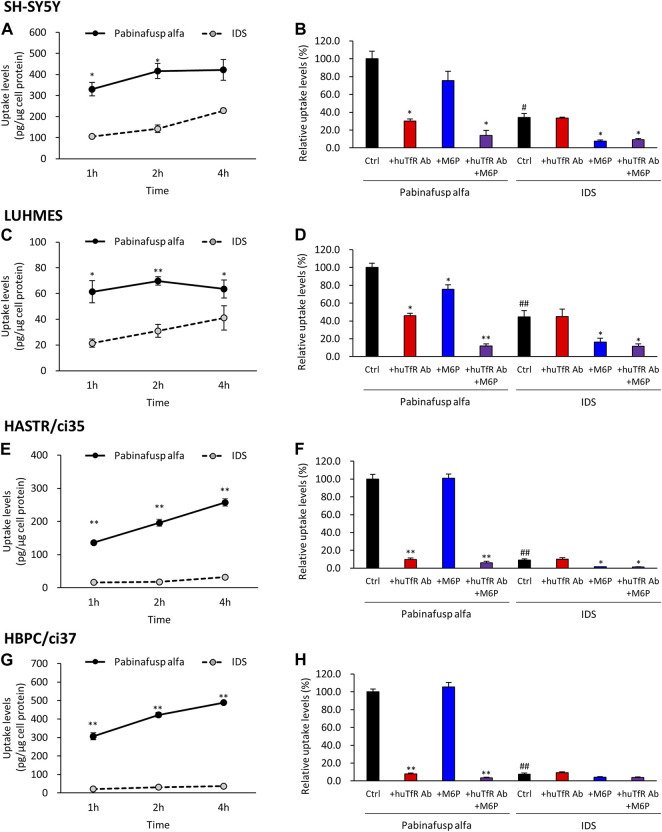
Characterization of pabinafusp alfa uptake profiles in various brain cell types. ^125^I-labeled pabinafusp alfa (1 μg/mL) or ^125^I-labeled IDS (1 μg/mL) uptake were examined in differentiated SH-SY5Y **(A, B)**, differentiated LUHMES **(C, D)**, HASTR/ci35 **(E, F)**, and HBPC/ci37 cells **(G, H)**. In **(A, C, E, G)**, the incubation (uptake) periods were set at 1, 2, and 4 h, and the uptake levels (pg/μg cell protein) of pabinafusp alfa (solid line) and IDS (dotted line) are shown as the mean ± S.E. of values obtained from three separate experiments (N = 3), each performed in duplicate (*, *p* < 0.05; **, *p* < 0.01; Student’s t-test). In **(B, D, F, H)**, the uptake levels were examined for 2 h in the presence or absence of huTfR Ab and/or M6P, which serve as competitive inhibitors for TfR and M6PR, respectively (huTfR Ab is the same anti-human TfR antibody as that used in pabinafusp alfa). Each data shows the relative uptake level (%) when calculating the pabinafusp alfa uptake levels without inhibitors (Ctrl) as 100%. Data are shown as the mean ± S.E. of values obtained from three separate experiments (N = 3), each performed in duplicate (*, *p* < 0.05; **, *p* < 0.01 vs. each of the Ctrl; ^#^, *p* < 0.05; ^##^, *p* < 0.01 vs. pabinafusp alfa Ctrl; one-way ANOVA followed by Student’s t-test). The data of each experiment are shown in Supplementary Figures S5A–8A.

In both cell types, significant pabinafusp alfa uptake activities were observed (Figures 4A,C). Additionally, we found that huTfR Ab, as well as M6P (to a lesser extent), partly inhibited pabinafusp alfa uptake, but that simultaneous exposure to these inhibitors totally eliminated the uptake (Figures 4B,D). In contrast, IgG did not affect the pabinafusp alfa uptake, and G6P only marginally inhibited the uptake probably because it may serve as a weak inhibitor of M6PR (Tong and Kornfeld, 1989) (Supplementary Figures S5B, 6B). Taken together, these results indicate that both receptors are effectively involved in pabinafusp alfa uptake in neuronal cells. On the other hand, IDS uptake was considerably inhibited by M6P, but not by the others (huTfR Ab, IgG and G6P), showing that M6PR plays an exclusive role in the IDS uptake in both SH-SY5Y and LUHMES cells (Figures 4B,D; Supplementary Figures S5B, 6B).

### Characterization of pabinafusp alfa uptake by human astrocytes and pericytes

In addition to neuronal cells, we also focused on astrocytes and pericytes. Astrocytes, which are another major brain cell type, cover BMECs with their endfeet, and pericytes exist in the immediate vicinity of BMECs. Their anatomical positioning motivated us to investigate whether astrocytes and pericytes can also capture pabinafusp alfa during its distribution into the brain. To this end, we conducted the pabinafusp alfa uptake assays utilizing human immortalized astrocyte and pericytes model cells, HASTR/ci35 and HBPC/ci37 cells. These cell lines were selected because we and other researchers have shown that they retain many of their original characteristics (Furihata et al., 2016; Kitamura et al., 2018; Umehara et al., 2018; Quan et al., 2020; Das et al., 2022).

The results of the uptake assays showed that both HASTR/ci35 and HBPC/ci37 cells possessed the pabinafusp alfa uptake capability (Figures 4E,G). The uptake activities were considerably inhibited by huTfR Ab, but not by IgG (Figures 4F,H; Supplementary Figures S7B, 8B), which are well consistent with the TfR expression in these cells (Figure 2). However, M6P did not apparently affect their overall uptake activities, and it slightly inhibited the uptake activities only when huTfR Ab was present (Figures 4F,H). Therefore, these results show that TfR is primarily involved in pabinafusp alfa incorporation into these cells.

Consistently, in the IDS uptake assays, the M6P-sensitive (namely, M6PR-mediated) IDS uptake levels were very low in both cell types (Figures 4F,H). Nevertheless, M6P appeared to attenuate the IDS uptake activities in these cells.

## Discussion

We here clarified the uptake processes of pabinafusp alfa in the human brain cells, which are well consistent with the hypothesis shown in Figure 1B. The findings that the TfR-targeting property of pabinafusp alfa plays a pivotal role in its incorporation into human BMECs, which become the first *in vitro* evidence, are well in line with the BBB penetration concept of the drug. The results also highlight the clear BBB permeability difference between pabinafusp alfa and IDS. Furthermore, identification of remarkable roles played by the TfR-targeting property of pabinafusp alfa in its incorporation into human neurons, astrocytes, and pericytes, will be valuable information that is sure to further understanding of the mechanisms underlying its pharmacological actions, as will be described below.

Turning to neuronal cells, it appears that TfR provides an additional route for the pabinafusp alfa uptake independent of the M6PR route. As the uptake results show, it can be presumed that pabinafusp alfa more effectively delivers IDS into neuronal cells than native IDS does, likely resulting in efficient removal and inhibition of GAG accumulation in neurons. This can be an important reason why pabinafusp alfa shows the clinical efficacy.

Furthermore, identification of significant levels of TfR-mediated uptake activities in astrocytes and pericytes is worth to be discussed. At first, it can be speculated that their high uptake levels might compromise pabinafusp alfa distribution to neurons. This cannot be fully excluded; however, we can also draw another hypothesis if an amount of pabinafusp alfa that enters the brain is large enough to provide its pharmacologically-relevant concentrations in both neuronal and astrocytes/pericytes. That is, the TfR-targeting strategy could allow pabinafusp alfa to accelerate IDS replacement not only in neuronal cells but also in astrocytes and pericytes in which GAG accumulation may also be related to CNS symptoms in MPS II patients (Kobolák et al., 2019; Maeda et al., 2019), thereby collectively facilitating global reduction and prevention of GAG accumulation in the brain. This, in turn, would provide a significant pharmacological advantage with the potential to have far-reaching effects on CNS symptoms in MPS II patients. Considering the clinical success of pabinafusp alfa, the hypothesis is presumed to be plausible, but in order to prove it, the relationship between uptake level and pharmacological potency of pabinafusp alfa needs to be investigated in future studies.

Regarding the results found in astrocytes and pericytes, it is difficult to see contribution of M6PR to the pabinafusp alfa uptake by HASTR/ci35 and HBPC/ci37 cells as if the receptor could not be functional. This does not seem to be due to the lack of the sufficient expression levels of the receptor, because the results of Western blotting analyses show that the M6PR protein expression levels are not so dramatically lower than those found in neuronal cells in which M6PR apparently participates in pabinafusp alfa and IDS uptake. Furthermore, based on the molecular size and anti-M6PR antibodies used in this study, M6PR detected in our cells should be the isoform supposed to be capable of extracellular lysosome enzyme uptake (Stein et al., 1987). Therefore, it is considered likely that the M6PR-mediated pabinafusp alfa uptake occurs in HASTR/ci35 and HBPC/ci37 cells as implied by the results showing that the IDS uptake levels are suppressed by M6P in these cells, but, in case of pabinafusp alfa uptake, the relative M6PR-mediated uptake levels are too small to be properly appreciated. Taken together, it is reasonable to believe that, like neurons, TfR, as well as M6PR (albeit its very low contribution), are functionally involved in pabinafusp alfa uptake in astrocytes and pericytes. The clear difference in their uptake levels may be explained by the possibilities: M6PR expression at the plasma membrane is not so abundant, or TfR leads to more efficient endocytosis.

Based on our results, it is intriguing to consider that equipment of another endocytosed property with ERT drugs may mark a previously unnoticed design concept in the new drug development for CNS diseases, including MPS II. Since we cannot say so categorically that TfR is the best of the best targets for enzyme delivery into the brain, it is worth exploring another better or unique endocytic receptor. New receptor-targeting bioengineering of therapeutic enzymes may provide an ingenious way to enhance broader brain distribution and thus give rise to stronger pharmacological actions. Therefore, in future studies, it will be necessary to identify and characterize certain targetable endocytic proteins in each human brain cell type.

Finally, we would like to briefly describe limitations of this study. It should be borne in mind that *in vitro* cell lines cannot always recapitulate *in vivo* characteristics due to their different survival microenvironments. For example, receptor expression levels of the cell lines may not be comparable those found *in vivo*. Pathophysiological conditions may also affect receptor expression levels *in vivo*, which is very difficult to reproduce *in vitro*. Therefore, careful consideration is necessary in interpretation of the results of this study. Nonetheless, it can be also true that the results are likely to, at least in part, show certain aspects that are relevant to what happens *in vivo*, which will be important clues for further studies on pabinafusp alfa or ERT drug development.

To summarize, our results clearly show that the TfR-targeting antibody moiety enables pabinafusp alfa not only to penetrate into human BMECs, but also into human neurons, astrocytes, and pericytes, which collectively suggests the possibility that the impressive clinical results of pabinafusp alfa can be attributed to cooperative effects of its BBB-penetration and its broader and deeper distribution into the brain. Interestingly, the TfR-targeting property of pabinafusp alfa may provide the first clinically successful example of the “killing two birds with one stone” strategy in the bio-design field of new modalities. Thus, our findings can also be viewed from the standpoint of not only shedding new light on efforts aimed at deciphering the pharmacological mode of action of pabinafusp alfa but also from the standpoint of paving the way for developing future drugs that fight against CNS symptoms of MPS II as well as other CNS diseases.

## Data Availability

The original contributions presented in the study are included in the article/Supplementary Material, further inquiries can be directed to the corresponding authors.
