# Supplementary material for: Transferrin receptor-targeting property of pabinafusp alfa facilitates its uptake by various types of human brain-derived cells in vitro
Source: Front Drug Deliv. 2023 Jul 3;3:1082672. doi: 10.3389/fddev.2023.1082672 (PMC12363330; doi:10.3389/fddev.2023.1082672)
Supplement: Supplementary file 1 [file DataSheet2.pdf]

Figure S1

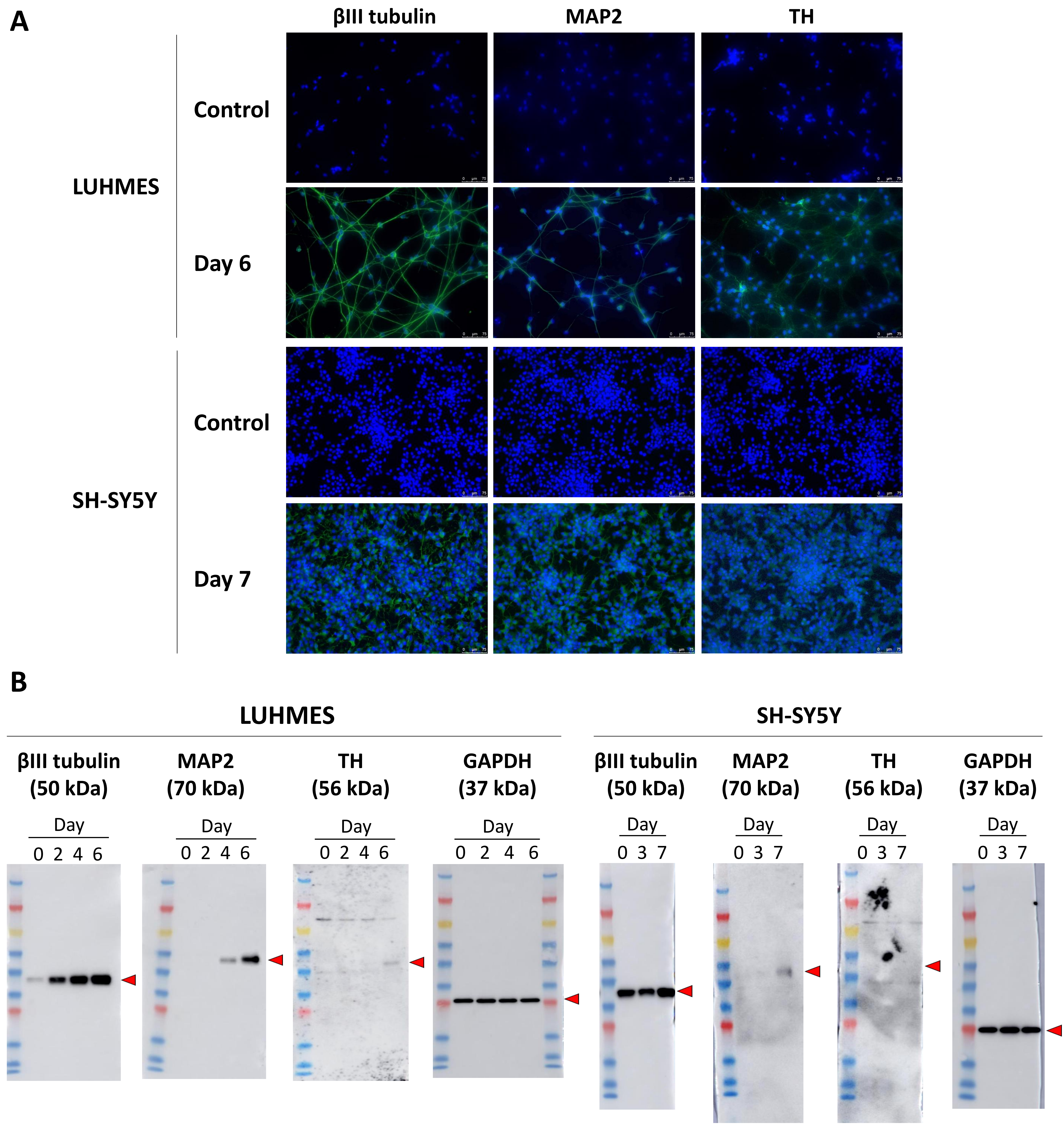

Supplementary Figure S1. Differentiation status of neuronal cell lines (LUHMES and SH-SY5Y) were validated by examining neuronal marker gene expression by immunocytochemistry (ICC) **(A)** and Western blotting analyses **(B)**. **(A)** Each cells were prepared with the same culture method used for the uptake assays and cultured for 6 (LUHMES) or 7 (SH-SY5Y) days. Targeted genes are βIII tubulin, microtubule-associated protein 2 (MAP2), and tyrosine hydrolase (TH). The images of the cells only treated by 2nd antibody are also shown as negative control. **(B)** Cell lysates from LUHMES cells (on day 0/2/4/6 of culture) and SH-SY5Y cells (on day 0/3/7 of culture) were prepared. Targets are the same with that used in ICC, and glyceraldehyde 3-phosphate dehydrogenase (GAPDH) was used as a loading control. The results obtained from a single experiment (N=1) are shown, and red arrows indicates the position where the bands would appear.

Figure S2

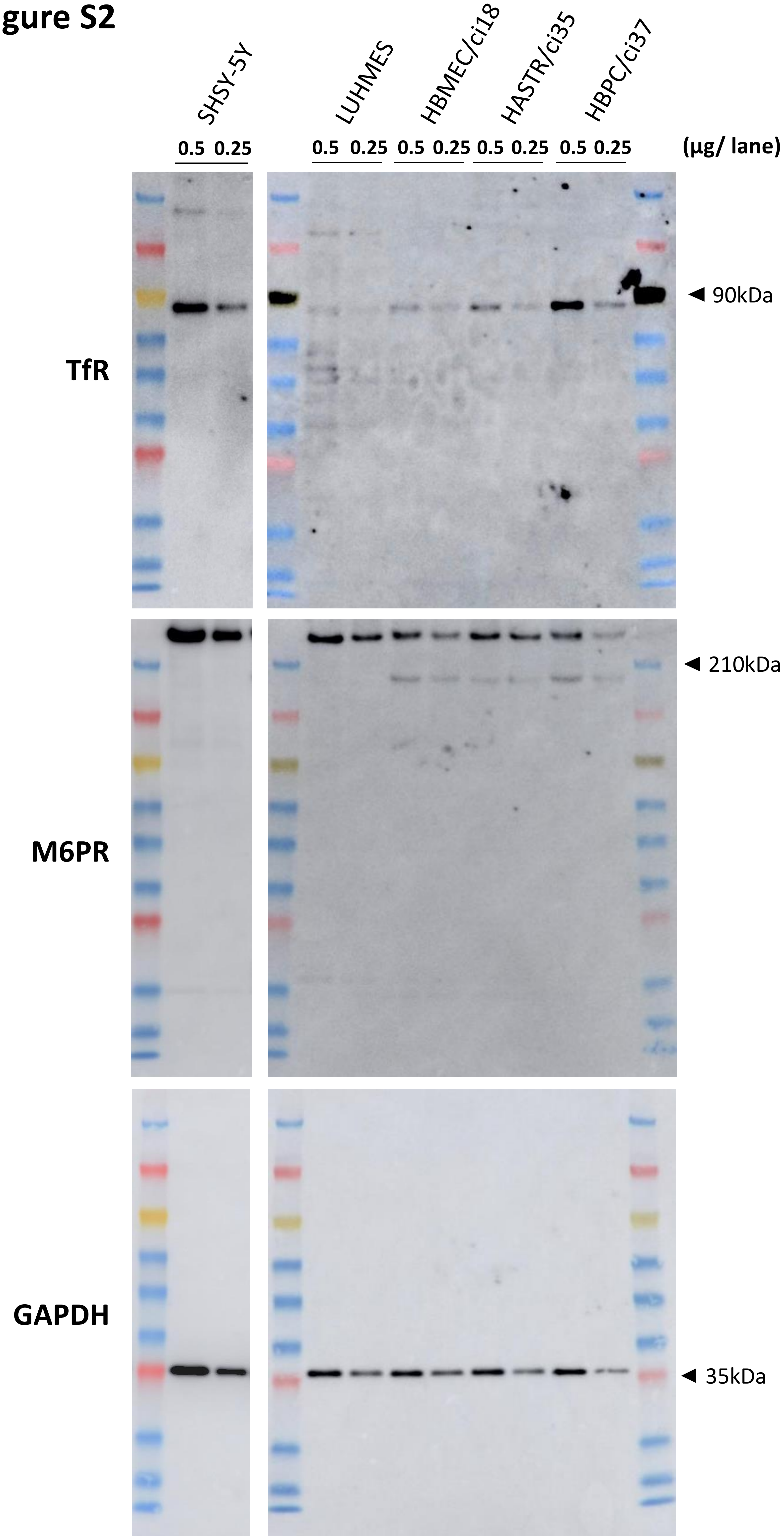

Supplementary Figure S2. Full, uncropped images of Western blotting analyses (Figure 2) detecting TfR, M6PR, and GAPDH proteins are shown.

Figure S3

A HBMEC/ci18

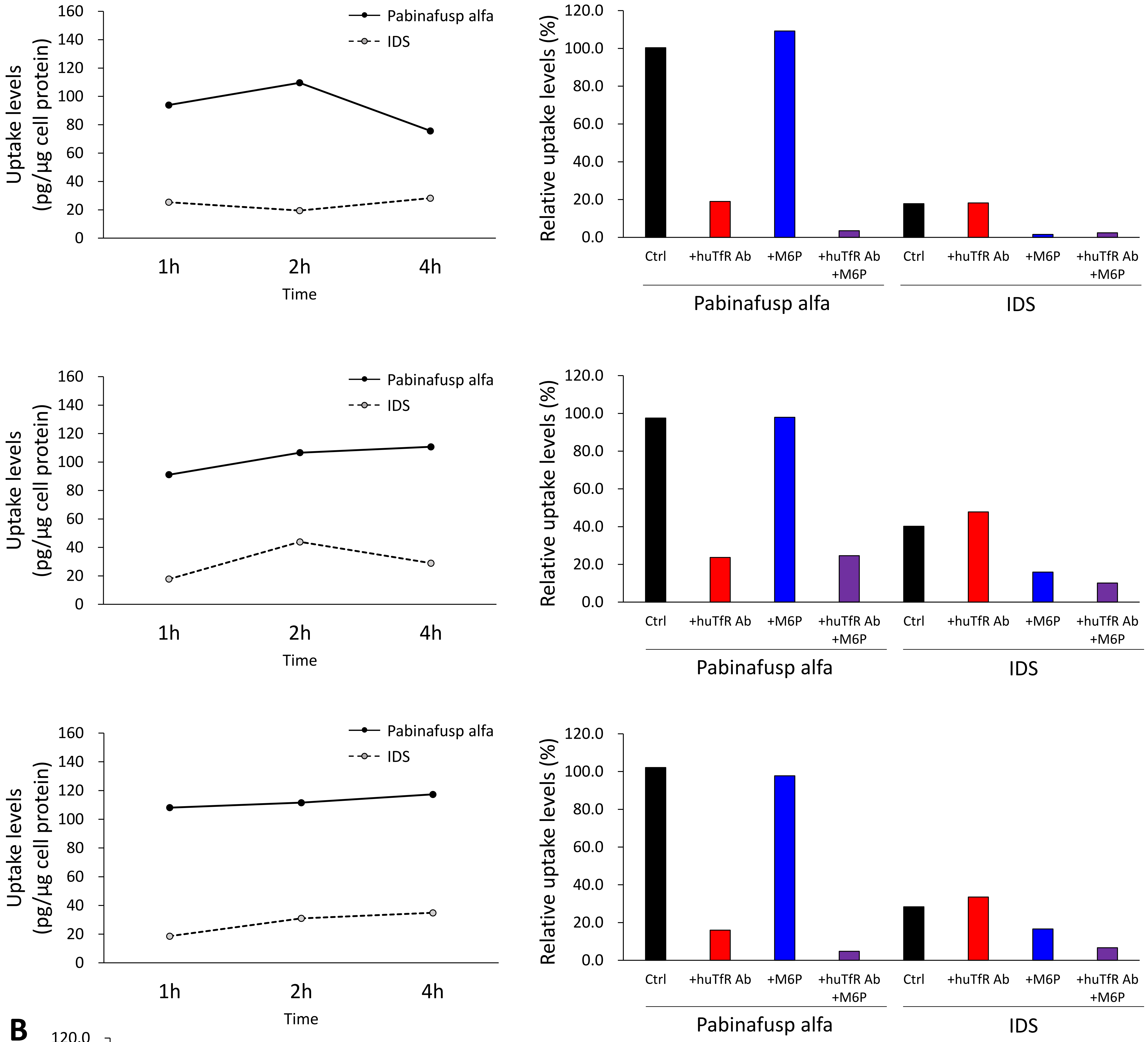

B

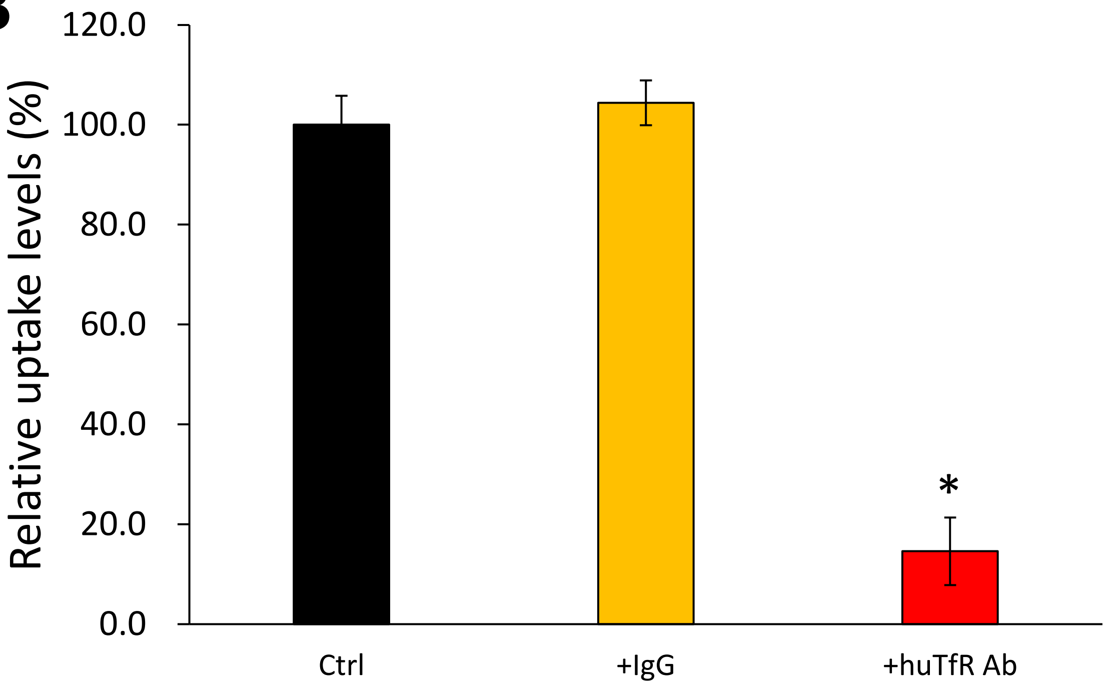

Supplementary Figure S3. (A) Results from three separate experiments (N=3) conducted for investigating uptake profiles of pabinafusp alfa and IDS in HBMEC/ci18 cells (Figures 3A and 3B) are shown. **(B)** Results of inhibition assays using huTfR or its isotype control IgG for pabinafusp alfa uptake in HBMEC/ci18 cells are shown. Data are shown as the mean ± S.E. of values obtained from three separate experiments (N=3), each performed in duplicate (*, $P < 0.05$ *vs.* Ctrl; Student's t-test).

Figure S4

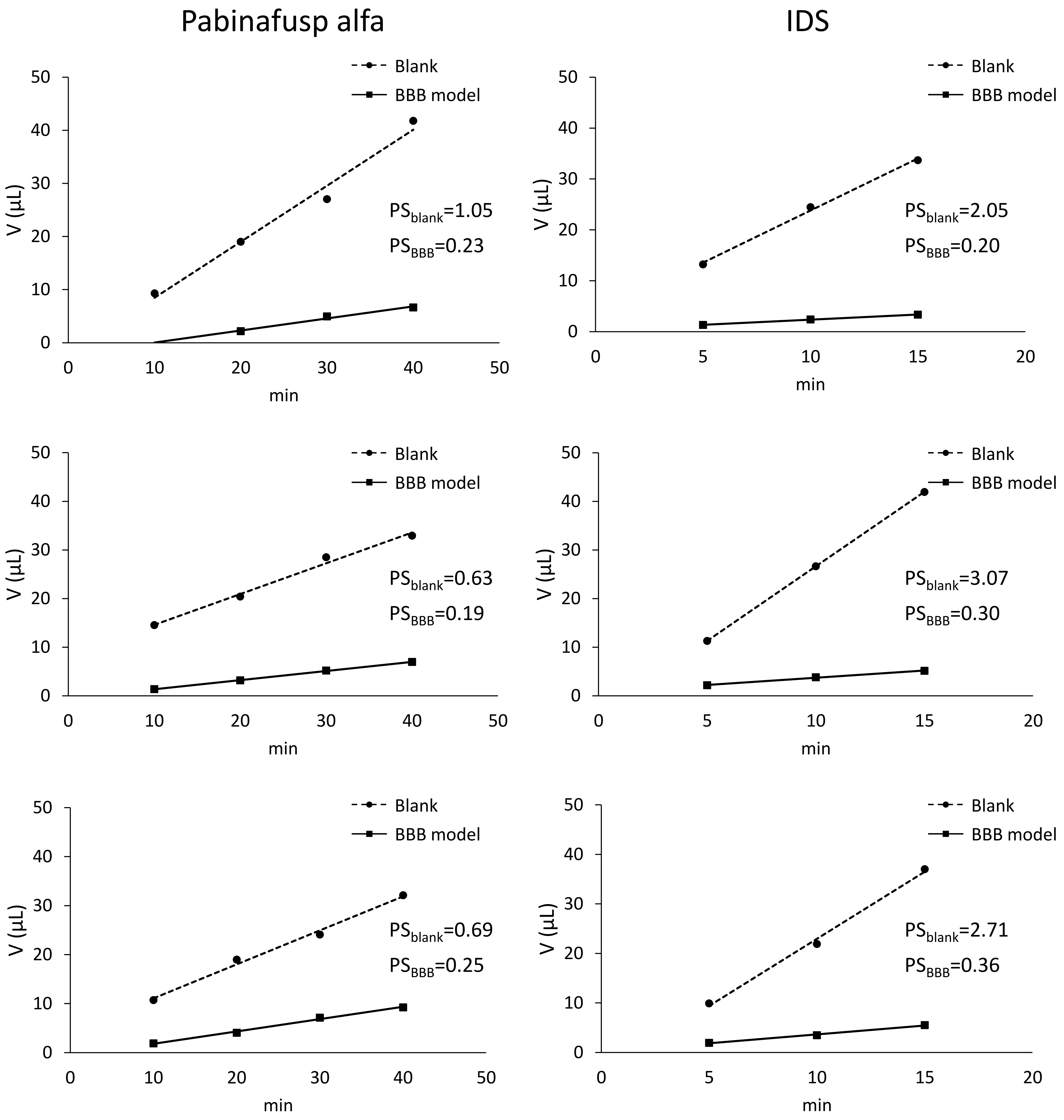

Supplementary Figure S4. Results from three separate experiments (N=3) conducted for investigating *in vitro* BBB permeability of pabinafusp alfa and IDS in HBMEC/ci18 monocultured BBB models (BBB model) or the models without the cells (blank) (Figure 3C) are shown. Permeability-surface area product values (PS, µL/min) obtained from the BBB model and the blank are indicated in as PS_{BBB} and PS_{blank}, respectively.

Figure S5

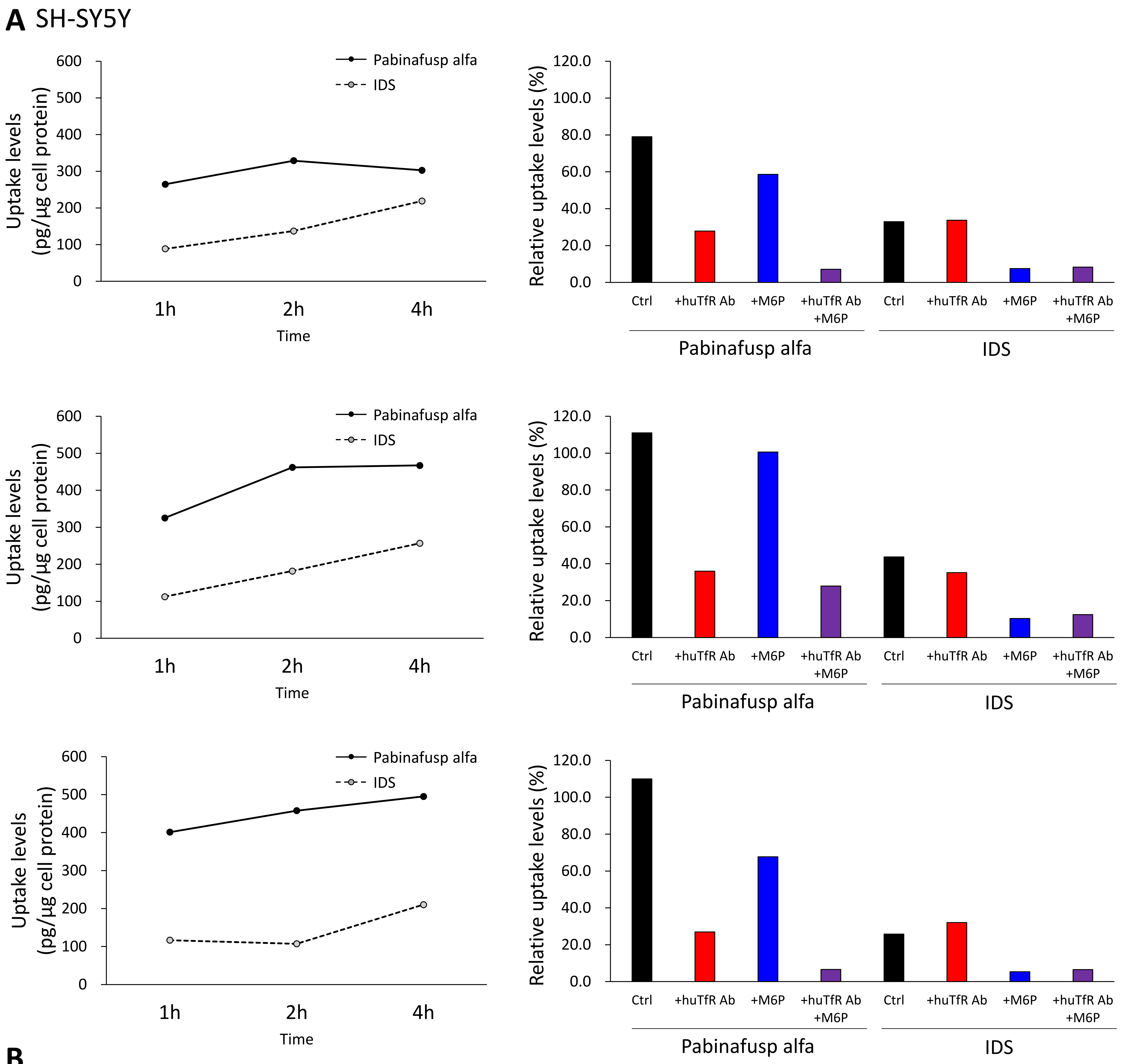

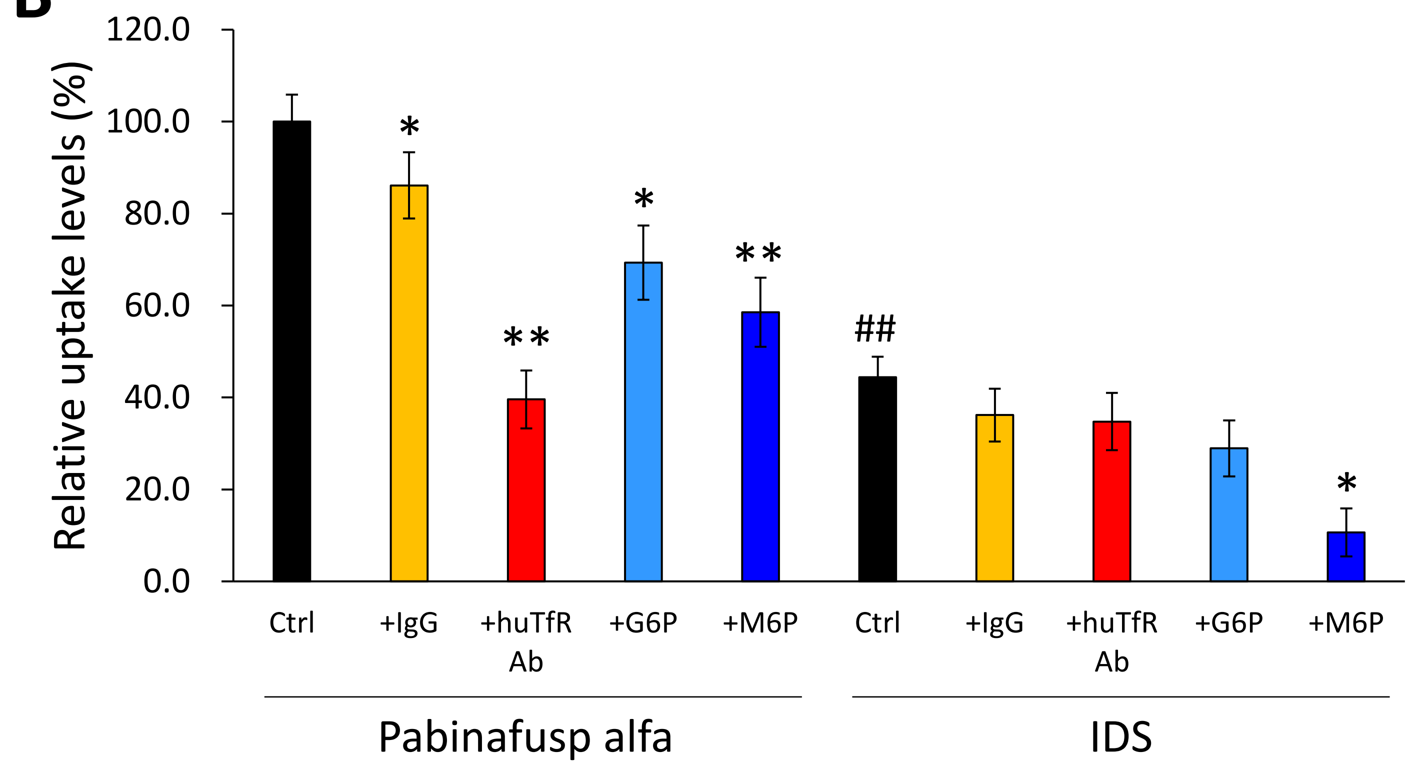

Supplementary Figure S5. (A) Results from three separate experiments (N=3) conducted for investigating uptake profiles of pabinafusp alfa and IDS in SH-SY5Y cells (Figures 4A and 4B) are shown. **(B)** Results of inhibition assays using huTfR, M6P and these negative controls (IgG for huTfR and G6P for M6P) for pabinafusp alfa or IDS uptake in SH-SY5Y cells are shown. Data are shown as the mean ± S.E. of values obtained from three separate experiments (N=3), each performed in duplicate (*, $P < 0.05$; **, $P < 0.01$ *vs*. each of the Ctrl; ##, $P < 0.01$ *vs*. pabinafusp alfa Ctrl; one-way ANOVA followed by Student's t-test).

Figure S6

A LUHMES

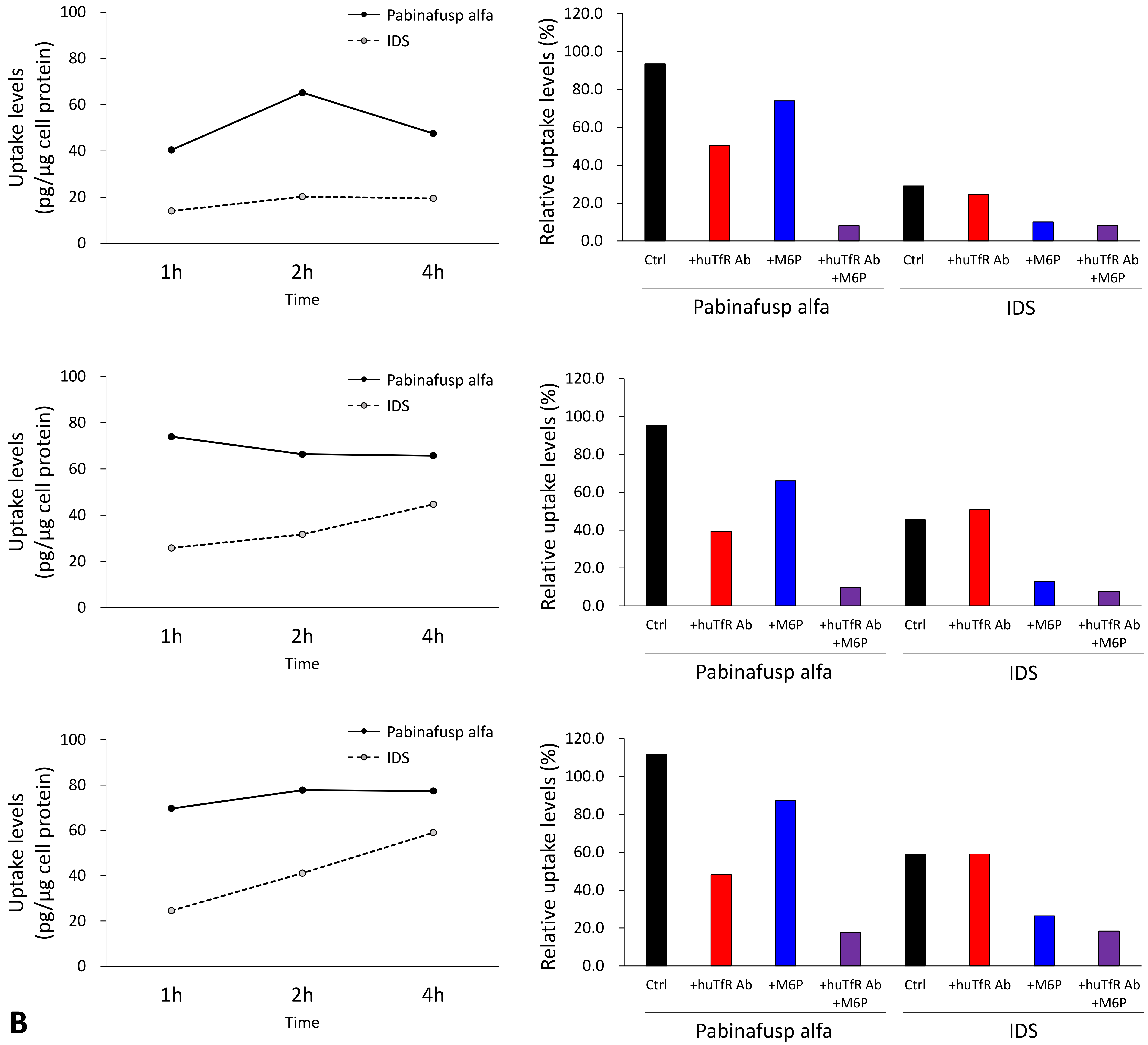

B

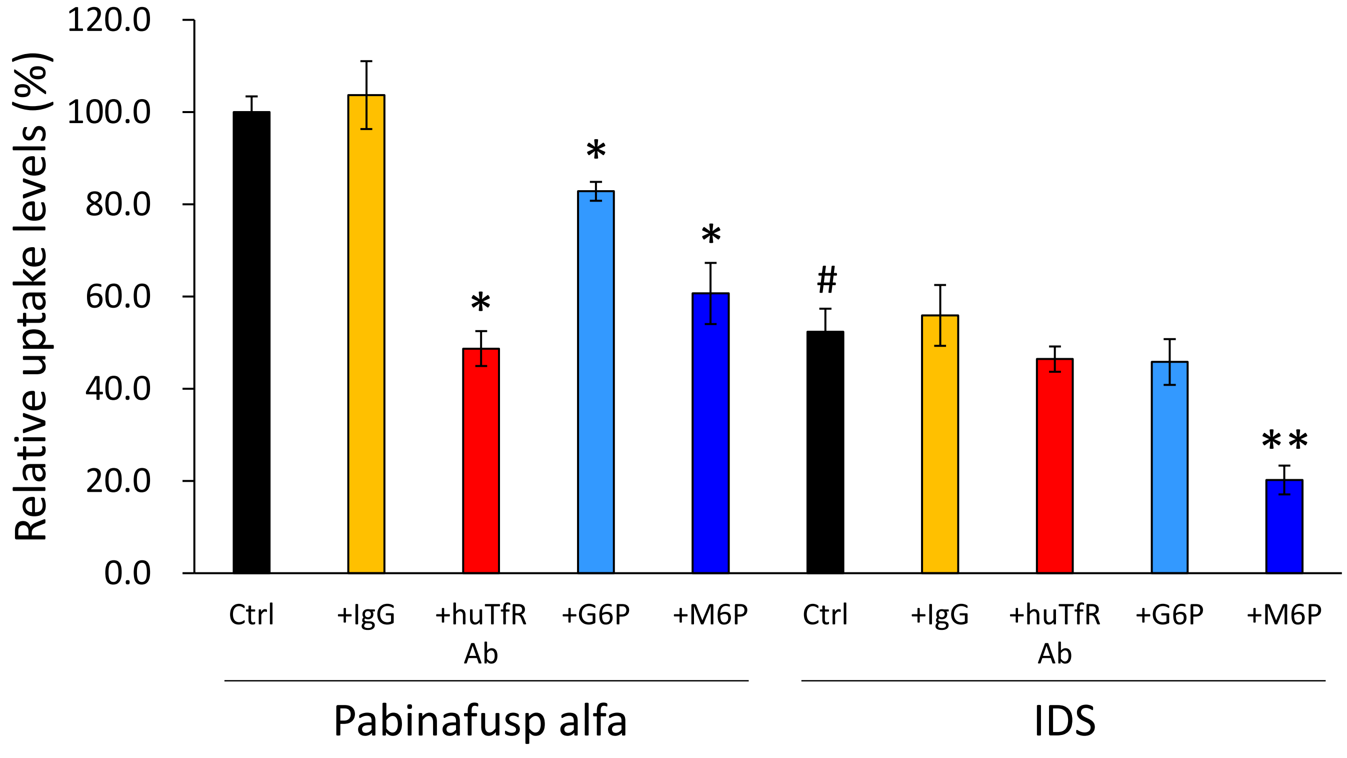

Supplementary Figure S6. (A) Results from three separate experiments (N=3) conducted for investigating uptake profiles of pabinafusp alfa and IDS in LUHMES cells (Figures 4C and 4D) are shown. **(B)** Results of inhibition assays using huTfR, M6P and these negative controls (IgG and G6P, respectively) for pabinafusp alfa or IDS uptake in LUHMES cells are shown. Data are shown as the mean ± S.E. of values obtained from three separate experiments (N=3), each performed in duplicate (*, $P < 0.05$; **, $P < 0.01$ *vs*. each of the Ctrl; #, $P < 0.05$ *vs*. pabinafusp alfa Ctrl; one-way ANOVA followed by Student's t-test).

Figure S7

A HASTR/ci35

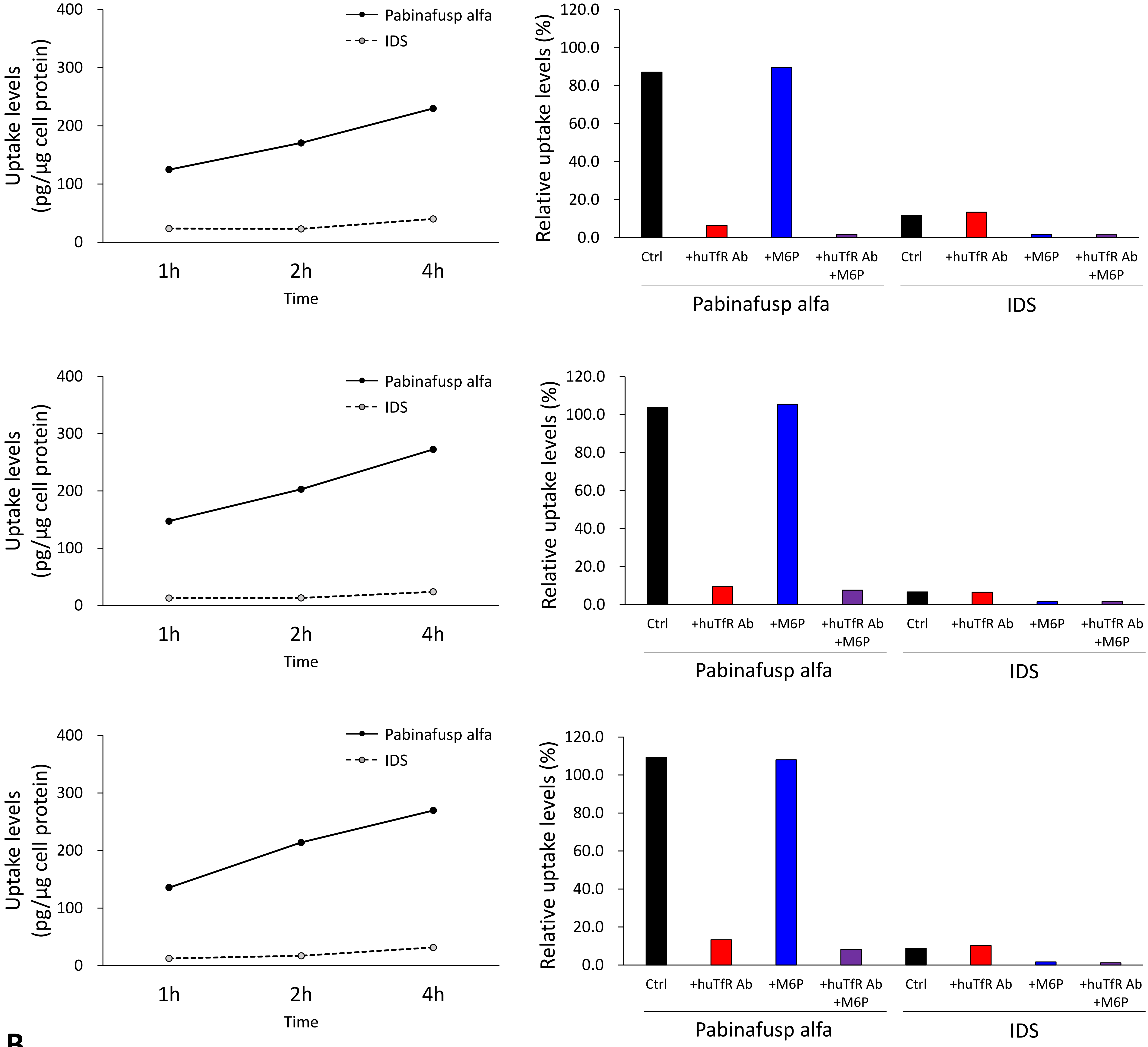

B

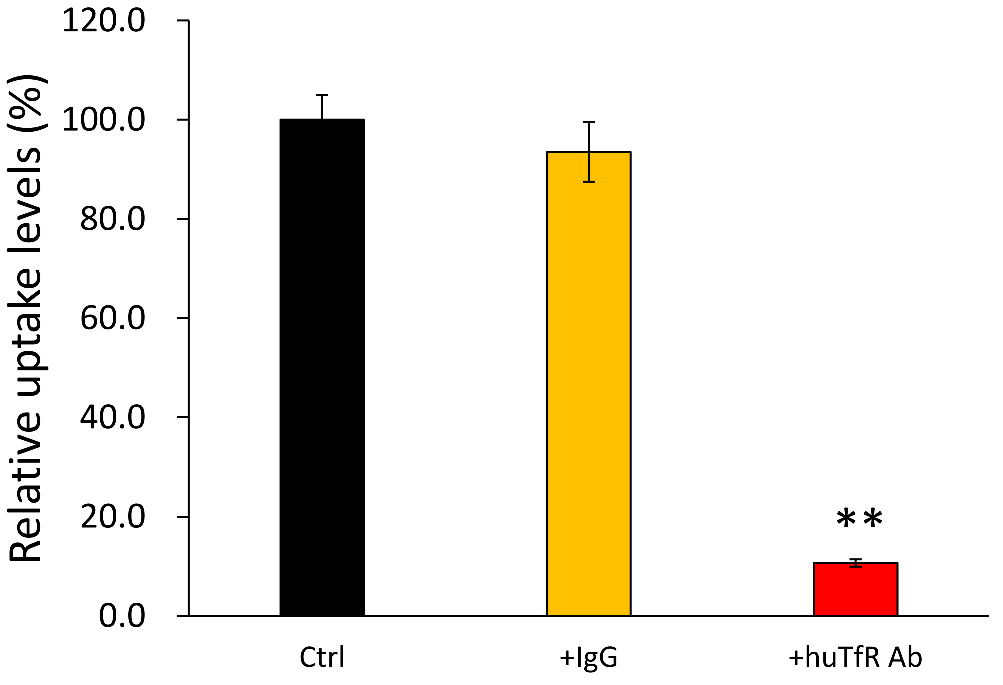

Supplementary Figure S7. (A) Results from three separate experiments (N=3) conducted for investigating uptake profiles of pabinafusp alfa and IDS in HASTR/ci35 cells (Figures 4E and 4F) are shown. **(B)** Results of inhibition assays using huTfR or its isotype control IgG for pabinafusp alfa uptake in HASTR/ci35 cells are shown. Data are shown as the mean ± S.E. of values obtained from three separate experiments (N=3), each performed in duplicate (**, P < 0.01 *vs.* Ctrl; Student's t-test).

Figure S8

A HBPC/ci37

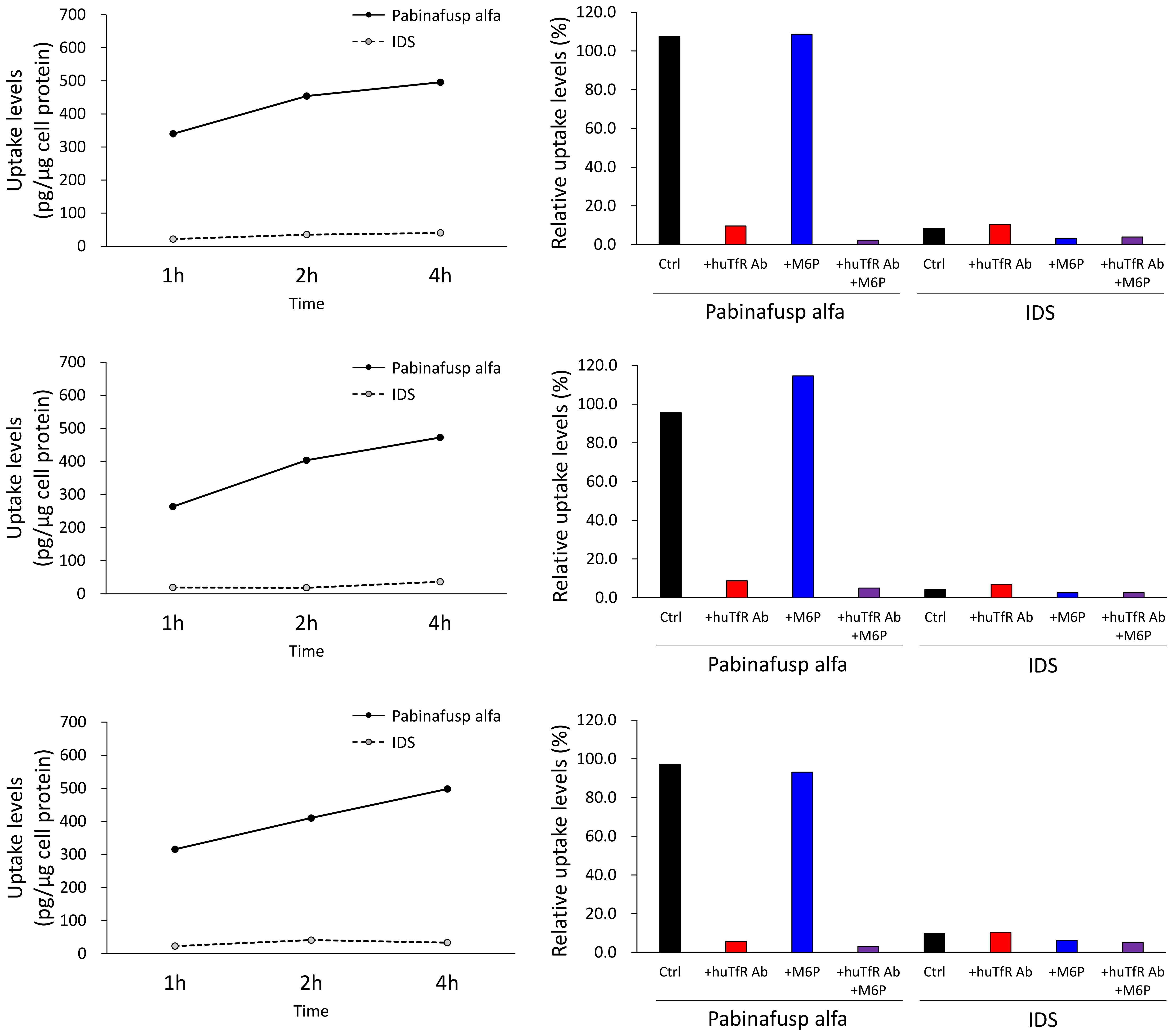

B

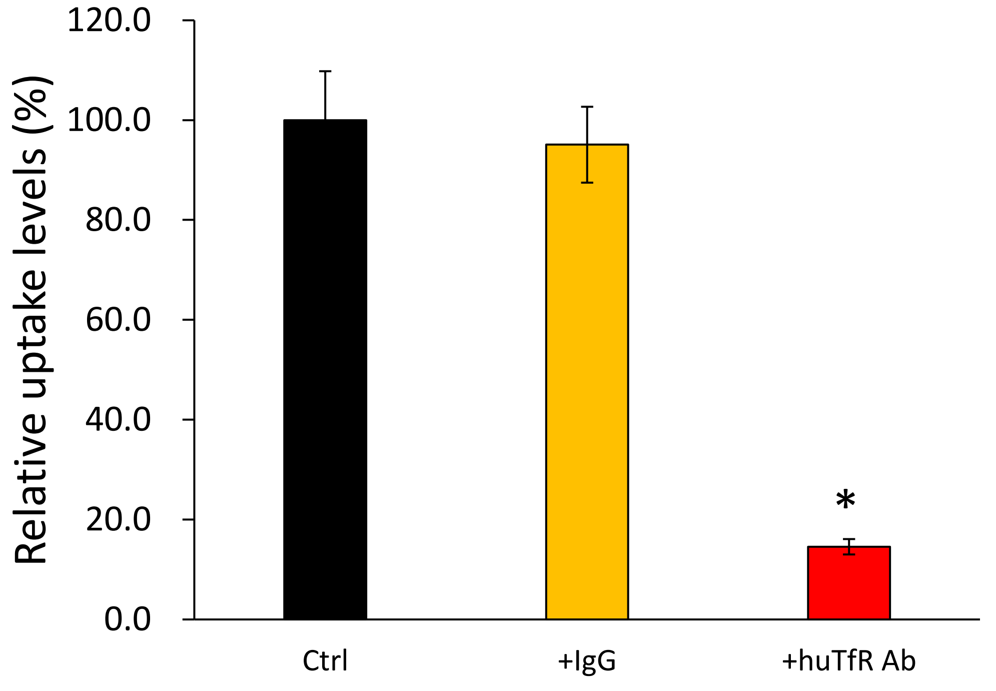

Supplementary Figure S8. (A) Results from three separate experiments (N=3) conducted for investigating uptake profiles of pabinafusp alfa and IDS in HBPC/ci37 cells (Figures 4G and 4H) are shown. **(B)** Results of inhibition assays using huTfR or its isotype control IgG for pabinafusp alfa uptake in HBPC/ci37 cells are shown. Data are shown as the mean ± S.E. of values obtained from three separate experiments (N=3), each performed in duplicate (*, P < 0.05 *vs.* Ctrl; Student's t-test).
